# Decisions in Motion: Decision Dynamics during Intertemporal Choice reflect Subjective Evaluation of Delayed Rewards

**DOI:** 10.1038/srep20740

**Published:** 2016-02-12

**Authors:** Denis O’Hora, Rachel Carey, Aoife Kervick, David Crowley, Maciej Dabrowski

**Affiliations:** 1 School of Psychology, National University of Ireland, Galway, University Road, Galway, Ireland; 2 Department of Clinical, Educational & Health Psychology, University College London, 1-19 Torrington Place, London WC1E 7HB, UK; 3 Insight Centre for Data Analytics, National University of Ireland, Galway, University Road, Galway, Ireland; 4 Department of Computing, Creative Media & Information Technology, Institute of Technology, Tralee, Dromtacker, Tralee, Co. Kerry, Ireland

## Abstract

People tend to discount rewards or losses that occur in the future. Such delay discounting has been linked to many behavioral and health problems, since people choose smaller short-term gains over greater long-term gains. We investigated whether the effect of delays on the subjective value of rewards is expressed in how people move when they make choices. Over 600 patrons of the RISK LAB exhibition hosted by the Science Gallery Dublin^*TM*^ played a short computer game in which they used a computer mouse to choose between amounts of money at various delays. Typical discounting effects were observed and decision dynamics indicated that choosing smaller short-term rewards became easier (i.e., shorter response times, tighter trajectories, less vacillation) as the delays until later rewards increased. Based on a sequence of choices, subjective values of delayed outcomes were estimated and decision dynamics during initial choices predicted these values. Decision dynamics are affected by subjective values of available options and thus provide a means to estimate such values.

Everyday, we are confronted by decisions that force us to trade off short-term against long-term consequences. Do you opt for a fast-food dinner, or a nutritious home-cooked meal? Should you spend money on an expensive jacket, or put it in a savings account? Will you smoke a packet of cigarettes, knowing they will harm your health? In each case, the short-term outcome, though smaller, exerts a stronger than expected influence on our decision. Such intertemporal decisions pose a personal challenge to our conceptions of ourselves as rational decision-makers. Too often, long-term benefits are sacrificed for short-term gain and the financial and health costs of such decisions pose grand challenges for society. The current study investigated whether patterns of movement during decision making are related to an individual’s relative evaluation of short-term and long-term consequences.

The effects of anticipated future consequences on our current behaviour are a function of the delay until those consequences will be experienced, an observation termed ‘delay discounting’. Given the personal and societal impact of delay discounting, it is unsurprising that there has been an awareness of the effects of delay on preference since the beginnings of economics (see^1^ for a review), such conflicts being noted by Adam Smith in *The Theory of Moral Sentiments* as early as 1759^2^. In attempting to quantify the relationship between delay and utility, Samuelson^3^ proposed that the subjective utility of a delayed consequences is discounted at an exponential rate, but behavioural research has typically indicated that individuals discount consequences at short delays more steeply than an exponential curve, while discounting later rewards less steeply. This sharp early decrease in utility is implicated in preference reversals in which individuals choose the later and larger of two rewards when both are distant (e.g., €11 in 31 days or €10 in 30), but they will reverse this preference as the two rewards get closer (€11 tomorrow vs €10 today). Consequently, the decrease of utility with delay is often better modelled using a hyperbolic or hyperboloid function with more than one parameter^4^.

Although it is clear that subjective utility decreases with delay, it is less clear how and why utility decreases as it does. That is, it is not clear how the interaction of cognitive and motivational processes gives rise to delay discounting. Neuroscientific studies have implicated a range of brain regions in intertemporal choice, as befits a complex class of decisions that has been analysed in a remarkable variety of procedures. Carter, Meyer and Huettel^5^ conducted a meta-analysis of fMRI studies using activation level estimation (ALE) and identified 25 reliable clusters of active coordinates including value-sensitive brain regions (medial prefrontal cortex, orbitofrontal cortex, ventral striatum and anterior insula) and regions involved in future planning and theory of mind (medial prefrontal cortex, inferior prefrontal cortex, temporal-parietal cortex, and peri-splenial posterior cingulate). More recent work has identified the dorsolateral prefrontal cortex as crucial in evaluating delayed choices^6^,^7^ and resisting the temptation of immediate rewards^8^,^9^. Nevertheless, the neural basis of the evaluation of intertemporal choices remains hotly contested. Summarising recent findings, Figner^10^ identified three broad classes of theories that are supported to varying degrees by neuroscientific evidence: single system “neurometric” theories, in which a single valuation system compares hyperbolically discounted options (e.g.,^6^), two system theories that include an “impatient” system sensitive to immediate reward and a “patient” system that discounts late options less steeply (e.g.,^11^), and self-control theories, in which reward valuations favouring an immediate option can be overruled by a goal-directed decision system (e.g.,^8^,^10^,^12^).

The foregoing neuroscientific research has highlighted candidate processes occurring during intertemporal choices. The motor execution of such choices provides a further source of information on the character of these decision making processes. The motor system does not wait for us to “make up our mind”; instead, when multiple courses of action are available to us, competition between these actions is expressed in the motor system^13^,^14^,^15^,^16^,^17^. When cognitive processes persist during motor responses, it is possible for characteristics of these processes to influence the ongoing performance of those actions^18^,^19^,^20^,^21^. The most reliable evidence of this influence is that, when required to make a motor response from an initiation point to the location of one of a set of options, response trajectories during decisions are more complex (i.e., are slower, deviate more from the shortest route and vacillate more) when the options are more similar, and trajectories are less complex when the options are more different. In one of the classic investigations of response trajectories, Song and Nakayama^22^ required participants to point to a lefthand option if a target number was less than 5 and to a righthand option if the target number was greater than 5. When target number was 4 (1 unit distance from 5), complexity of trajectories was highest, and complexity reduced as the target number decreased from 4 to 1. Similar patterns were observed for numbers greater than 5 towards the right-hand option. Thus, when options are more similar, cognitive competition between options persists longer before a choice is determined. This cognitive competition may be expressed in motor competition when a person is in motion during the resolution of a choice.

A number of researchers have exploited this interaction between cognitive and motor activity to explore characteristics of decision making^23^,^24^. In True/False information-based decisions, Dale and colleagues^25^ demonstrated that the crowd-based probability of confirming an item predicted the average trajectories of mouse cursor responses choosing True and False. As the probability of confirmation reduced from 1 (always confirmed) to zero (always denied), trajectories of computer mouse responses exhibited the expected degree of complexity in terms of velocity of the response, deviation towards the unchosen option, and vacillation during the response. Direct trajectories towards True were observed for probabilities close to 1, more complex trajectories (i.e., slower, more deflected and with more vacillations) toward True for lower probabilities above 0.5, more complex trajectories toward False for probabilities close to but below 0.5 and direct trajectories towards False for probabilities close to 0. Koop and Johnson^26^ extended this work in the area of preferential choice and demonstrated that the disparity in the pleasantness of emotionally-valenced images (International Affective Picture System) predicted deviation during mouse cursor trajectories, with greater valence disparities (very pleasant versus very unpleasant) giving rise to more direct trajectories. In a second experiment, trajectories while choosing risky gains (least direct) and safe losses exhibited greater deviation than safe gains (most direct) and risky losses. The foregoing studies demonstrate that when subjective values of available options are more similar, cognitive competition persists longer and gives rise to more complex trajectories. In the case of Dale and colleagues, crowd-sourced probabilities of affirmation provided approximations of the relative subjective evaluations of True and False for a particular statement. When the crowd-sourced probability of True is 0.8, then relative subjective value of True in the presence of that statement is approximately 0.8. Even though participants may have varied in their particular evaluations, the average relative subjective values (i.e., the crowd-sourced probabilities) predicted the average trajectories. Similarly, Koop and Johnson employed crowd-sourced emotional valences of images as approximations of the relative subjective values of the images when participants are required to choose between them.

In the case of delay discounting, it is well established that delay reduces the subjective value of future rewards. However, the degree to which such delays affect value differs across individuals. Indeed, the degree to which an individual discounts future rewards is interpreted as a measure of impulsivity, with individual differences in delay discounting linked to higher rates of obesity (e.g.^27^), sexual risk-taking (e.g.^28^,^29^), substance abuse (see^30^ for a review), and additional pathologies such as gambling or impulsive behaviours in clinical populations^31^,^32^. Consequently, the effects of delay on the subjective value of available options vary across individuals, such that more impulsive individuals will be more attracted to rewards at shorter delays than less impulsive individuals. Scherbaum, Dshemuchadse and colleagues^33^,^34^ tracked computer mouse response trajectories during two studies of intertemporal choice. In order to deal with varying effects of delay across individuals, they controlled for the effect of delay at the level of the individual and identified difficult choices as those in which the individual’s subjective values of available rewards were more similar and easier choices in which the subjective values of available rewards were more different. As the difference in subjective values of the presented options reduced, complexity of response trajectories increased.

The current study employed a different procedure from Scherbaum, Dshemuchadse and colleagues that enabled us to make more specific predictions of subjective value from response trajectories. To individualise the estimate of relative subjective value of a future reward, we examined responses across a series of decisions and inferred the amount of immediate money that was equally attractive as that future reward (see section B of Fig. 1). This “indifference point” provides an estimate of the subjective value of the future reward (e.g., if the probability of choosing €16 in a week against €12 now is 0.5, then €16 in a week is worth €12). Specifically, we employed a staircase procedure in which intertemporal decisions were presented in sets^35^ at a number of prescribed delays. The reward available now was adjusted across decisions in order to estimate the subjective value of the future reward in terms of reward available now. This procedure allowed us to investigate a number of specific research hypotheses by tracking computer mouse movement during binary decisions. First, given that cognitive competition is reduced as the difference in subjective values increases and that delay decreases the subjective value of future rewards, the response dynamics of choosing immediate rewards should become less complex (i.e., faster, less deflected and with fewer vacillations) as the delay until future alternatives increases. Second, one would expect that the subjective value of the delayed reward in the first decision at a delay should influence both the dynamics of responding in the first decision and responses to further decisions. Consequently, it should be possible to predict subsequent choices at a delay based on features of computer mouse movement during the initial choice at that delay. The staircase procedure employed in the current design is particularly suited to addressing this hypothesis. Of particular interest is whether decision dynamics allow improved prediction of subjective value over prediction based on delay alone. Finally, we investigated these effects for both monetary decisions and decisions about favoured rewards (“vices”) to assess whether such effects are observed across delayed hypothetical rewards.

The current study brought the action dynamics of intertemporal choice out of the laboratory and into a public setting, the Science Gallery Dublin (https://dublin.sciencegallery.com/), a science museum in Dublin city, Ireland. As part of an exhibition in May 2013 called RISK LAB^36^ that focused on the mathematics and psychology of risky choices, patrons were asked to choose from a set of vices (alcohol, food, smoking or “none of the above”) and then completed a short series of intertemporal choices, during which their mouse cursor trajectories were collected. Participants who chose one of the available vices (i.e., alcohol, food or smoking) completed 20 monetary decisions followed by a further 20 decisions between amounts of their preferred vice now or at a delay. Participants who did not choose one of the available vices (i.e., they chose “none of the above”) completed participation following the 20 monetary decisions. Decisions were completed in sets of 4 at a set delay (e.g., 1 week, see Fig. 1). Participants first made a decision between €8 Now and €16 at the specified Delay. For this and each subsequent decision, if the participant chose the Future option (i.e., €16 at the specified Delay), then the Now option increased in value for the next decision (e.g., choose between €12 Now and €16 at the specified Delay). If, however, the participant chose the Now option (e.g., €8 Now), then the next Now option decreased in value (e.g., choose between €4 Now and €16 at the specified Delay). The delayed option was always €16, so, by varying the money Now, we estimated the point of subjective equality between the Now option and the Future option (i.e., how much €16 at the specified Delay was worth in terms of money Now). This design enabled us to estimate delay discounting very quickly (within 20 decisions; between 2 and 5 minutes) as required in a public setting.

It is worth noting that, in the current study, participants were required to choose between hypothetical rewards at hypothetical delays. This “hypothetical” approach has been adopted in a majority of delay discounting studies^37^ and was adopted here due to the experimental context of a short discounting game in a public environment. Even though responses to hypothetical future rewards may differ to responses to real rewards delayed in real time, there are well established practical, financial and ethical factors^38^ that support using hypothetical rewards, which were particularly pertinent given this particular context.

## Results - Monetary Decisions

Delay discounting was observed in the allocation of choices by participants. Future options were less likely to be chosen (i.e., reduced in value) as delay increased. In the left-most panel of Fig. 2, the average net present value (NPV; subjective value in terms of immediate money) of €16 at a delay (ie., its subjective value in terms of immediate money) is plotted as a proportion of €16 (e.g., if €16 in a week had a subjective value of €8 Now, it was plotted as 0.5). The degree of discounting (*k*) was estimated using the area under the empirical discounting function (Area Under the discounting Curve; AUC) method proposed by Myerson, Green, and Warusawitharana^39^. This estimate of discounting (*k* = 1−AUC) requires few assumptions about the shape of the relationship between Value and Delay and is typically less skewed than other measures of discounting. Descriptive statistics indicate that, in line with previous research (e.g.,^40^), participants who chose smoking as their vice discounted money most strongly (*M* = 0.68, *SD* = 0.23; see Fig. 2, rightmost panel). Participants who did not choose a vice discounted money least strongly (*M* = 0.59, *SD* = 0.28). However, there was no significant difference in the AUC-based *k* measure (*F* 3,621 = 1.24, *p* = 0.29) across Vice groups.

### The Dynamics of Choosing between Short-Term and Long-Term Gains

A core question in the current study was whether a participant’s decision dynamics were related to the subjective value of the available choices. As a first investigation of this possibility, three indices of the decision dynamics were isolated from the choice trajectories. Response time was taken as the time from initiation of a decision to the clicking of a choice option. Curvature was measured in terms of maximum deviation, the point of maximum perpendicular distance in the choice movement from the straight line from start of a choice to its completion. This variable was bimodally distributed, so it was dichotomised into wide (maximum deviation greater than 500 pixels) and tight trajectories. Finally, vacillation was measured in terms of changes in the x direction or “x flips”^41^.

**Figure 1:**
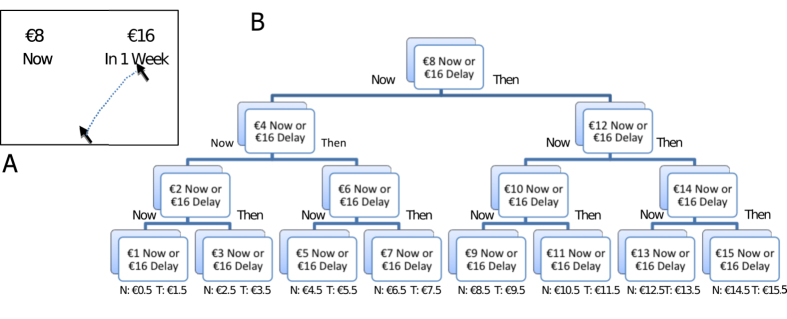
Representation of the experimental design. (**A**) For the Monetary Decisions, participants began by choosing between €8 Now and €16 at one of five delays (e.g., 1 week; see text for details). Cursor mouse movement was recorded during choosing. (**B**) Based on their choices, participants were presented with further choices that attempted to more precisely quantify the participant’s subjective evaluation of €16 at the delay in terms of money now. For example, if a participant chose €16 in 1 week over €8 Now, we know that the subjective value of €16 in 1 week is greater than €8 Now, so the participant is offered a choice between €16 in 1 week and €12 Now. Trajectories observed in the first choice predicted the pattern of subsequent choices made by a participant. In Experiment 2, euro amounts were replaced with amounts of a vice chosen by the participant (e.g., 8 bottles of beer now or 16 bottles of beer in 1 week).

**Figure 2:**
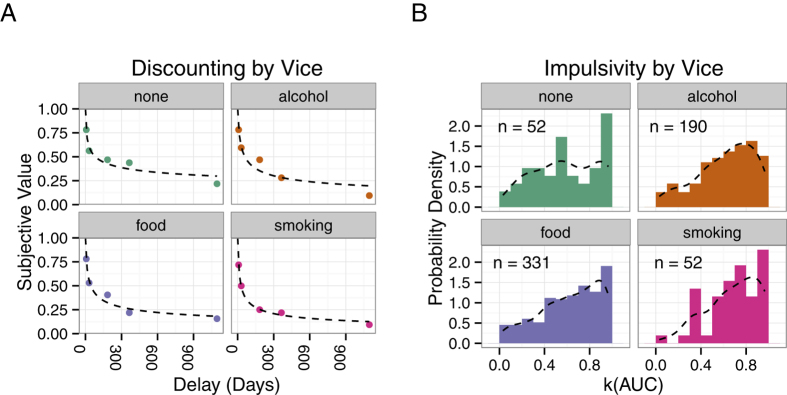
Delay discounting of Monetary Decisions. (**A**) In the plots on the lefthand side, the median subjective value of the Future option (e.g., €16 in a Week) relative to €16 Now is plotted against the delay to receiving that reward for each Vice group. As delay increases, there is a nonlinear decrease in subjective value. The curve is the best fitting hyperboloid function^52^,^53^. (**B**) In the righthand panel, in order to depict the range of impulsivity across vice groups, histograms of values of *k* based on the area under the discounting curve are presented for each vice group and a probability distribution curve is overlaid. All plots were designed using ggplot2^54^.

**Figure 3:**
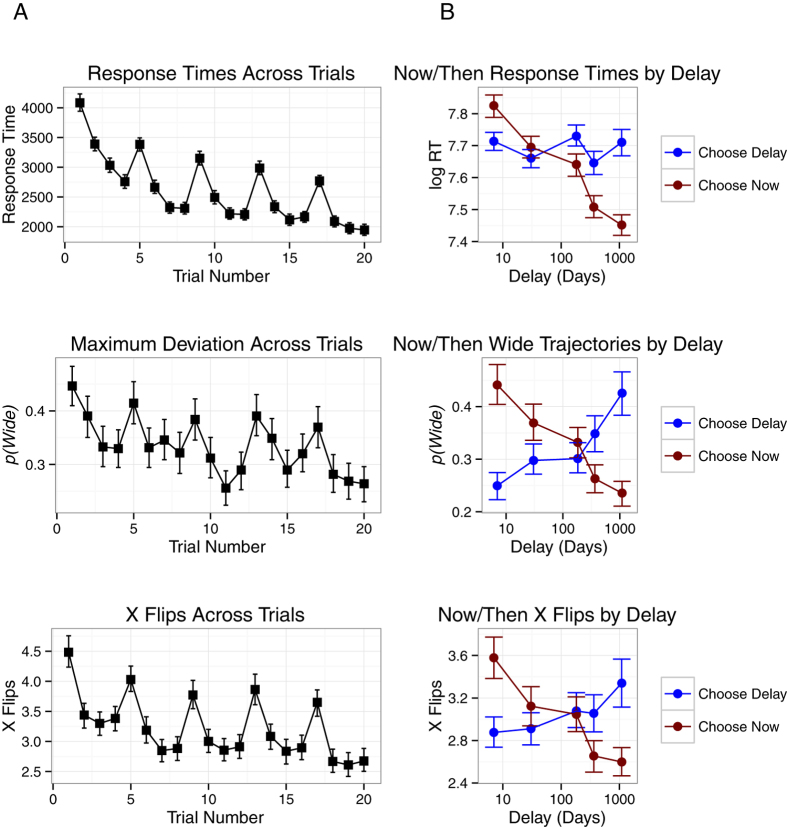
The effects of Decision Number and Delay on mouse cursor trajectory measures in Monetary Decisions. (**A**) The lefthand column presents the change across decisions in three measures of mouse cursor trajectories (Response Time, Maximum Deviation and X Flips). (**B**) The righthand column presents the change in the same trajectory measures with Delay. Error bars represent bootstrapped confidence intervals.

Mixed effects models were employed to assess statistical significance of effects^42^,^43^; a linear model was employed to predict response time, a binomial model to predict wide or tight trajectories and a poisson model to predict number of x flips. In all choices, participants chose between an amount of money now (the Now option) and a greater amount at a delay (the Future option). As delays increase, the subjective value of the Future option should decrease. Thus, it was expected that, as delays increased, trajectories towards Now choices would be faster, less deflected and include fewer vacillations (an interaction between Delay and the option chosen). Variables in the model to assess the central research question included Delay and Choose Now (whether the chosen option was the Now option or the Future option). Three further control variables were also included: Demographics, Decision Number and First Choice. Demographics refers to the presence of demographic data (see Method for details). Decision Number was included in order to capture any practice effects with a session, as all three indices reduced as a function of the number of decisions completed (see lefthand plots in Fig. 3). First Choice refers to the first decision at a delay; this can be seen in Fig. 3 as spikes in trajectory measures on the first, fifth, ninth, thirteenth and seventeenth decisions. To briefly recap, decisions were completed in sets of 4 at a set delay (e.g., 1 week, see Fig. 1); Participants first made a decision between €8 Now and €16 at the specified Delay and then the Now option increased (if the participant chose the Future option) or decreased (if the participant chose the Now option) to identify the point of subjective equality between the Now option and the Future option. The First Choice variable was included in the model to control for this source of variability.

Decision trajectories with response times greater than 5 seconds or more than 10 x flips were excluded from analyses (8.6% of decisions). For all three decision movement indices, response trajectories towards the Now option were less complex for long delays and towards the Future option were less complex for short delays. This outcome supports the position that decision dynamics are affected by the relative subjective values of the available alternatives. When options are closer in subjective value, cognition competition increases and decision dynamics are more complex. Conversely, when options move further from one another in subjective value, competition reduces and decision dynamics are simpler. As Delay increased, the Future option reduced in subjective value and choosing the Now option was faster (*b*_RT_ = −0.0496, *p* < 0.001), was less likely to follow a wide trajectory (*b*_MD_ = −0.2978, *p* < 0.001) and incurred fewer vacillations (*b*_XF_ = −0.0596, *p* < 0.001; see Fig. 3). Interestingly, significant main effects suggested, regardless of Delay, choosing the Now option was faster on average (*b*_RT_ = −0.0224, *p* < 0.001), but more likely to be wide (*b*_MD_ = 0.1800, *p* < 0.001).

Participants who did not complete demographic information did not respond differently from those who did (see Table 1). The remaining two controls captured significance variance. Practice effects were observed across all three decision dynamics indices. Across decisions, choices became faster (*b*_RT_ = −0.1648, *p* < 0.001), wide trajectories were less likely (*b*_MD_ = −0.1876, *p* < 0.001) and vacillation was less likely (*b*_XF_ = −0.0731, *p* < 0.001). In each set of 4 choices at a delay, the first choice exhibited greater complexity; response times were longer (*b*_RT_ = 0.2719, *p* < 0.001), wide trajectories were more likely (*b*_MD_ = 0.4324, *p* < 0.001) and more left-right vacillations (x flips) were observed (*b*_XF_ = 0.2318, *p* < 0.001). One explanation of the increased complexity of responding to the first choice at a delay is that the subjective value of the Future reward was calculated during the first choice at a delay and remembered in subsequent decisions. It is also possible that increased complexity resulted from the presentation of novel delay information; the first choice required the integration of more information than the following choices.

### Decision Movement and Subjective Value

The previous results support the position that the difficulty of a decision (i.e., the similarity in value of the available options) may be reflected in the participants’ mouse movements during choice. As larger rewards became more delayed, the subjective value of those rewards decreased and participants chose the earlier option faster, more directly and with fewer vacillations. It is also clear that the first choice at a given delay induced the greatest complexity in decision dynamics, even though the values of the Now options should, on average, have been closer to the subjective value of the Future option in later choices. This is due to the staircase procedure adjusting to provide novel Now options closer to the subjective value of Future option.

**Figure 4:**
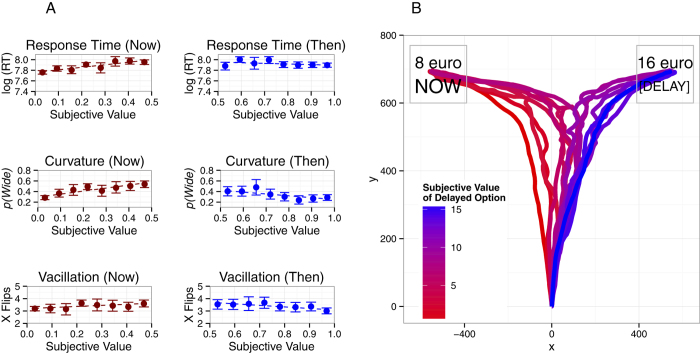
The effect of Subjective Value on mouse cursor trajectory measures during the first Monetary Decision at a delay. Subjective Value of €16 at a delay was calculated based on the participants’ responses across a series of four decision. (**A**) The leftmost column presents the relationship between Subjective Value and Response Time, Maximum Deviation and X Flips for sets of decision in which the participant first chose €8 Now. Subjective Value is reported in terms of €16, so 0.5 infers a a Subjective Valuation of €8. The next column from the left column presents the relationship between Subjective Value and the same trajectory measures for sets of decision in which the participant first chose €16 at a delay. Error bars represent bootstrapped confidence intervals. (**B**) The image on the righthand side depicts average trajectories (see text for details) of Monetary Decisions during the first decision at a delay grouped by the Subjective Value of the Future option (inferred from the set of four decisions at that delay) relative to €16 Now (the equivalence point). On all these measures, clear cut decisions, in which Subjective Values are high and low, exhibit simpler decision dynamics and complexity increases for moderate Subjective Values.

To assess whether decision dynamics during the first choice predicted subjective values inferred following the fourth choice (i.e., the final choice in a set), we plotted decision dynamics indices during first choices as a function of these subjective values (Fig. 4, Section A). Two columns of plots are provided; data from participants who chose the Now option are presented in the lefthand column, since these participants valued €16 at the specified delay lower than €8. Participants who chose the Future option treated €16 at the specified delay as more valuable than €8 and their values are presented in the righthand column. When participants chose Now, greater response complexity indicated higher subjective valuations of the Future option. That is, when the participant preferred the €8 Now to the Future option, greater complexity predicted higher subjective values that were less than, but approaching €8. When participants chose the Future option (subjective values of the Future option greater than €8 now), greater response complexity indicated lower subjective values of the Future option that were greater than, but approaching €8.

To further explore the dynamics of initial choice at a delay, average time-normalized trajectories (see Fig. 4, Section B) were estimated using the procedures summarized by Dale, Kehoe and Spivey^44^. Each trajectory was interpolated into 101 time steps. Using this approach, temporal information is lost, but the ordinal pattern of trajectory coordinates is preserved. Trajectories of decisions to the left-hand choice (Now and Future choice locations were counterbalanced across participants) were reflected in the vertical axis at the origin so that all trajectories ended at the right-hand choice. Then, responses to the Now choice were reflected such that Now choices were depicted as finishing in the top left and Future choices were depicted as finishing in the top right. As described earlier, if participants chose the €8 Now option, the subjective value of €16 at that delay was less than €8 and if participants chose €16 at that delay, the subjective value of €16 at that delay was higher than €8. Consequently, all the trajectories for each equivalence point finished at the same point (Now if <€8, Future if >€8; cf^25^).

Decision trajectories exhibited the expected modulation of decision dynamics by subjective value. That is, when the Future option was later estimated as having a low subjective value (e.g., €16 at a long delay), participants chose the Now option more directly. As the Future option increased in subjective value, trajectories toward the Now choice became increasingly complex until the value was above €8, at which point participants chose the Future option. Subjective values that were above, but close to, €8 were preceded by complex trajectories towards the Future option but as subjective values increased, complexity reduced. When the Future option was later estimated as having a high subjective value (e.g., €16 at a short delay), participants chose the Future option more directly. Notably, similar to previous findings^25^,^33^,^45^, trajectories were not symmetric, but rather exhibited a pronounced attraction toward the greater amount (€16). In this study, the greater monetary value of the Future option seems to have exerted an early attraction on choice trajectories (cf.^33^).

To analyze whether movement indices reflect subjective evaluation of choices at a delay, we employed linear mixed effects models to predict the equivalence point (i.e., the subjective value of the Future option in terms of euros now) derived from the fourth decision at a delay. To capture the known nonlinear effect of delay on subjective value, the log of Delay was employed as a predictor. The models thus assessed whether response time, dichotomised maximum deviation and x flips predicted the subjective value of the Future option over and above Delay. Separate models were employed to assess movement during choices of the Now option and choices of the Future option (Table 2). The log of Delay was expected to negatively predict subjective value of Now and Future choices (i.e., delay reduces subjective value). It was expected that movement indices would positively predict subjective value when participants chose €8 now and negatively predict subjective value when participants chose €16 at a delay. For participants who chose €8 now, the Future option (i.e., €16 at a delay) was worth subjectively less than €8, so higher equivalence points (i.e., closer to €8) would result in greater complexity. In contrast, for participants who chose €16 at a delay, the Future option was worth subjectively greater than €8, so higher equivalence points (i.e., further from €8) would result in lower complexity.

For participants who chose €8 now, both response time and dichotomised maximum deviation predicted subjective values of €16 at a delay in the expected direction, but x flips did not. For participants who chose €16 at a delay, only dichotomised maximum deviation predicted subjective values of €16 at a delay in the expected direction. In both analyses, demographic controls did not have a significant effect and log Delay predicted subjective value as expected.

### Decision Movement and Impulsivity

The foregoing analyses suggest that movement during a decision reflects the subjective evaluation of the options available. It is also possible that this evaluation reflects impulsivity, which may be estimated using *k*^45^. To assess whether impulsivity *k* was related to movement indices, we calculated correlations between *k* and response time, maximum deviation and x flips for the first choice at each of the five delays (Table 3). Raw maximum deviation was used instead of the dichotomised variable previously used in order to calculate correlations that were comparable across indices. We focused on the first choice at a delay, which was always between €8 and €16, to control for the effects of money. We distinguished between participants who chose the Now option and the Future option because, similarly to the relationships between movement indices and subjective value, we expected that more impulsive individuals would choose Now options more quickly and directly, and Future options more slowly with greater curvature. Therefore, negative correlations between *k* and movement indices were expected when participants chose the Now option (greater *k* predicts lower complexity) and positive correlations when participants chose the Future option (greater *k* predicts higher complexity). Finally, we calculated separate correlations for each delay in addition to calculating averages of trajectory measures across delays. We included each delay because mean trajectory measures across delays were likely to include high and low trajectory measures across delays within Now and Future options (e.g., a participant might have a deflected trajectory towards €8 Now compared to €16 in 1 year, but a direct trajectory towards €8 Now compared to €16 in 3 years) and such patterns would suppress correlations.

When considering these correlations, it is important to remember that different numbers of participants chose the Now and Future options at each level of Delay. In general, as Delay increased, more participants chose the Now option and fewer participants chose the Future option. Consequently, we present correlations for each Delay to control for the different distribution of Delays across Now and Future choices. The number of participants affects the probability value obtained for the size of correlation observed (see for example the correlations between *k* and response times at 3 years). Here, we will focus on significant correlations but full details of the observed relationships are available in Table 3.

Sporadic correlations were observed between *k* and response times. The *k* index correlated positively with both response times of both Now and Delay choices when choosing between €8 now and €16 in 1 week; thus, impulsive participants were slower to choose either option. When the choice was between €8 now and €16 in 3 years, *k* correlated negatively with time taken to choose Now options, suggesting that impulsive participants were quicker to choose the Now option. The remaining significant correlations with response time were as expected, with impulsive participants faster to choose €8 now over €16 in 6 months or €16 in 1 year.

Correlations between *k* and the maximum deviation of trajectories were largely in line with expectations. In the main, more impulsive individuals chose Now options more directly, and Future options less directly. Significant negative correlations were observed when participants chose the Now option when choosing between €8 now and €16 in 6 months or greater and significant positive correlations were observed when participants chose the Future option when choosing between €8 now and €16 in 1 year or less, except for choices at a delay of 1 month. Correlations between *k* and x flips were weaker than those observed between impulsivity and the other trajectory indices. One significant negative correlation was observed when participants chose the Now option when choosing between €8 now and €16 in 1 year. The variable correlations observed likely reflect a weakness of the short design based in a real-world setting, in that each participant made only one response at each delay and thus it was not possible to average out extraneous variance within participants as one might in a laboratory-based paradigm.

## Results - Vice Decisions

At the beginning of the study, participants were asked to choose one vice from a choice of alcohol, cigarettes, food or none of the above. If participants chose “none of the above”, then their participation concluded following the set of 20 monetary decisions reported above. If participants chose a vice, then they completed a further 20 decisions between amounts of their vice now and at a delay (e.g., 8 bottles of beer now or 16 bottles of beer in a week). In the main, participants responded similarly to these vice decisions as they had to the monetary decisions.

**Figure 5:**
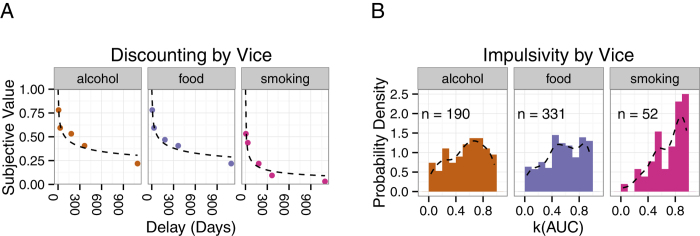
Delay discounting of Vice Decisions. (**A**) In the plots on the lefthand side, the median subjective value of the Future option (e.g., 16 bottles of beer in a Week) relative to 16 bottles of beer Now is plotted against the delay to receiving that reward for each Vice group. The curve is the best fitting hyperboloid function. (**B**) In the righthand panel, histograms of values of *k* based on the area under the discounting curve are presented for each vice group and a probability distribution curve is overlaid.

Similarly to the monetary decisions, delay discounting of vices was observed; Future options reduced in value as delay increased. The left-most panel of Fig. 5 presents discounting curves obtained for vice decisions. In line with previous research^40^, smokers (*M* = 0.71, *SD* = 0.23) discounted cigarettes more heavily than the alcohol (*M* = 0.55, *SD* = 0.27) and food (*M* = 0.57, *SD* = 0.27) vice groups discounted their respective vices. The effect of vice on *k* was significant (F2,570 = 7.4043, *p* = 0.0006) and helmert contrasts indicated no difference between the food and alcohol groups (*b* = 0.02135, *SE* = 0.02444, *t* = 0.873, *p* = 0.3828) but a significant difference between smokers and the other two vice groups (*b* = 0.04975, *SE* = 0.01306, *t* = 3.808, *p* = 0.0002).

In the analyses of decision dynamics, trajectories with response times greater than 5 seconds or more than 10 x flips were again excluded from analyses (6.3% of decisions). Effects were similar across movement indices, as was previously observed for monetary decisions (Table 4). As Delay increased, choosing the smaller sooner option was faster (*b*RT = −0.0445, *p* < 0.001), was less likely to follow a wide trajectory (*b*MD = −0.2637, *p* < 0.001) and incurred fewer vacillations (*b*XF = −0.0596, *p* < 0.001). In the main effects, decision trajectories towards the Now option were more likely to be wide (*b*MD = 0.1800, *p* < 0.001) as observed with monetary decisions, but there was no main effect on response time by the option chosen (*b*RT = 0.0105, *p* = 0.3331). For vice decisions, there was a main effect on vacillation by the option chosen; choosing the Now option exhibited significantly more vacillation (*b*XF = 0.0384, *p* < 0.001) than choosing the Future option.

The effects of the control variables replicated those seen with monetary decisions. First decisions at a delay exhibited longer response times (*b*RT = 0.2521, *p* < 0.001), higher probability of wide trajectories (*b*MD = 0.5555, *p* < 0.001) and more left-right vacillations (x flips) (*b*XF = 0.2454, *p* < 0.001). Two of the three indices reduced as a function of the number of decisions completed. Across decisions, responses became faster (*b*RT = −0.1471, *p* < 0.001) and vacillation was less likely (*b*XF = −0.0775, *p* < 0.001) but the probability of wide trajectories was relatively consistent across decisions (*b*MD = −0.0608, *p* = 0.0607), though more common for first decisions (see Fig. 5).

**Figure 6:**
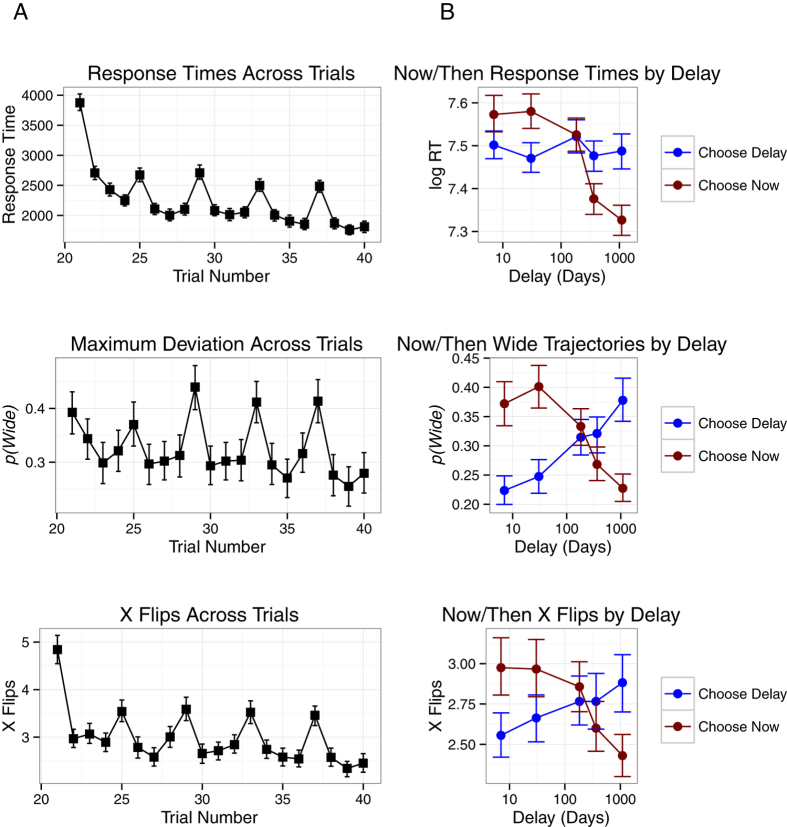
The effects of Decision Number and Delay on mouse cursor trajectory measures in Vice Decisions. (**A**) The lefthand column presents the change across decisions in three measures of mouse cursor trajectories (Response Time, Maximum Deviation and X Flips). (**B**) The righthand column presents the change in the same trajectory measures with Delay.

**Figure 7:**
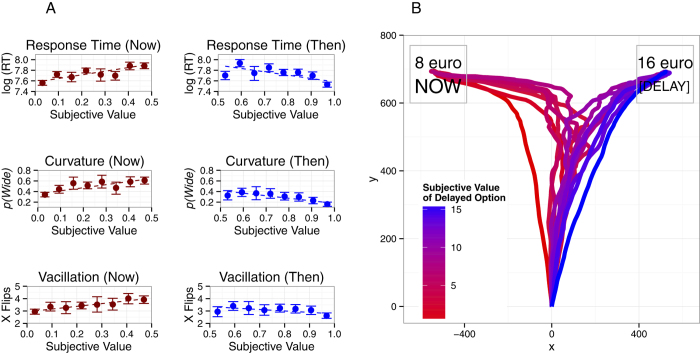
The effect of Subjective Value on mouse cursor trajectory measures during the first Vice Decision at a delay. (**A**) For both lefthand columns, movement indices are highest for moderate subjective values of the Future option, when that option was closest in Value to the Now option. (**B**) Average trajectories of Vice Decisions exhibit the same pattern. For more detail on these measures, see Fig. 4.

Subjective value affected trajectories of Vice decisions similarly to those of monetary decisions (see Fig. 6 and Table 5). When subjective value of the Future option was lowest, participants chose the Now option more quickly, more directly and with less vacillation. These patterns are observed in the movement indices and average decision trajectories depicted in Fig. 7.

Statistical analyses were conducted to assess whether movement indices during the first decision predicted the subjective value of the Future option. As for monetary decisions, decisions in which participants chose the Now option and the Future option were analysed separately. In both analyses, demographic controls did not have a significant effect and log Delay predicted subjective value as expected. For participants who chose the Now option, increases in response time (*b* = 0.0172, *p* = 0.0001) and wide trajectories (*b* = 0.0192, *p* = 0.0159) predicted increased subjective values of the Future option, but the number of x flips did not (*b* = 0.0079, *p* = 0.0557) though this latter effect was marginal. The opposite relationships were observed for participants who chose the Future option. Decreases in response time (*b* = −0.0227, *p* < 0.001) and the probability of a tight trajectory (*b* = −0.0208, *p* = 0.0077) predicted increased subjective values of the Future option, but number of x flips did not (*b* = 0.0047, *p* = 0.2184).

Relationships between vice decision dynamics and impulsivity fit better with expectations than the relationships observed with monetary decision dynamics (Table 6). In general, more impulsive participants chose Now options more quickly, more directly and with less vacillation and Future options less quickly, less directly and with greater vacillation. Significant negative correlations were observed between response time and *k* when participants chose the Now option in decisions between 8 units of their vice (e.g., 8 bottles of beer) now and 16 units of their vice in 6 months or greater. Significant positive correlations were observed when participants chose the Future option in decisions between €8 now and €16 in 1 month or 1 week. Thus, more impulsive individuals chose Now options more quickly, and Future options less quickly within these limits. In addition, more impulsive individuals chose Now options more directly when the Future option was in 6 months or longer, and Future options at delays of 1 week, 1 month or 3 years. Negative correlations were observed between vacillation and *k* when participants chose the Now option over delays of 6 months or 1 year. The improved correlations were likely due to a reduction in variability of responding, since the vice decisions were presented after the monetary decisions.

## Discussion

We sometimes make assumptions about others’ confidence in their evaluations on the basis of the manner in which they make choices. The current findings highlight the information available in the actions of decision-makers about their evaluations. Under conditions in which available options were of similar subjective value (i.e., both options close to €8 or 8 units Now), decision-makers’ actions were more complex; they were slower, more diverted from the shortest path to their eventual choice and vacillated more. Once quantified in an initial decision, these features of decisions predicted the subjective value of Future options based on subsequent decisions.

Decision making can be usefully divided into two types, perceptual and preferential decision making. Perceptual decisions require an individual to make a decision about perceptual phenomena such as whether a visual event occurred (“did I see something?”), whether that event was different in degree (e.g., strength, latency) than another event (“was the left one bigger than the right?”) or whether the event satisfied membership of an expected class (“did I see a circle?”). For such decisions, we can control much of the stimulation and responses to such stimulation are relatively similar across individuals. Research on the dynamics of motor movement during such decision supports the position that the motor system has access to the subjective value of options prior to completion of a decision^20^,^21^. In contrast, preferential decisions ask an individual to decide which of two events they prefer and such decisions are subject to complex sources of influence and often differ across individuals. One strength of the current paradigm was that we were able to infer the subjective evaluations of Future options based on an individuals’ set of decisions at a delay and then assess decision dynamics as these evaluations changed. Previous work on decision dynamics during preferential decisions has depended on crowd-sourced probabilities^25^, generally accepted quantitative differences^22^,^46^ or learned differences^24^,^26^,^47^,^48^ to infer differences in subjective value across conditions. The current experiments were able to capture the interaction between participant and condition (i.e., delay) to provide a more nuanced relationship. The effects of Delay on subjective value varied across individuals (i.e., the degree of discounting) and yet the relationship between decision dynamics and subjective value was robust.

Modern technologies enable us to capture detailed information of movement trajectories during decisions. However, response time remains the dominant measure of cognitive processes and it is not always clear how additional trajectory information (e.g., deflection and vacillation) adds to our understanding of cognitive processes. At the theoretical level, tracking movement has undermined a conception of decisions as discrete irreducible outcomes and promotes an understanding of decisions as extended interactions between an organism and its context. Decision makers take advantage of incomplete evaluations to begin responding prior to certainty^19^,^21^, meaning that evaluation information is available to the motor system during evaluation and not solely at the end of a decision. When presented with novel information during a decision, the brain takes account of the physical costs of changing one’s mind^49^ demonstrating that motor information affects evaluations. At a practical level, the current experiment highlights that the deflection of a decision towards the unchosen option provides information on the relative subjective values of the available options that is independent of response time information. Also, deflection measures during decisions correlated more closely with overall impulsivity than response time measures.

In a recent investigation of the action dynamics of intertemporal choice, Dshemuchadse and colleagues^33^ previously identified a greater attraction to non-chosen short-term smaller rewards than non-chosen long-term larger rewards. Such a bias fits well with explanations of delay discounting in terms of impulsivity. In the current study however, we observed a reverse bias towards larger rewards rather than sooner rewards. There are numerous differences between the paradigms employed in these two studies. For instance, Dshemuchadse *et al.* employed a laboratory-based design using many more decisions than we could ask our participants to make. Dshemuchadse *et al.* also presented amount and delay information consecutively, but we presented both types of information simultaneously. However, perhaps the most important difference between the paradigms was that the trajectories depicted in the current study were for choices (the first at each delay) in which the smaller reward amount (e.g., €8) was always 50% of the larger reward amount (€16), whereas Dshemuchadse *et al.* presented various amount ratios (the Future option ranged from 101% of the Now option to 500%). Moving initially towards the higher value option, as observed in the current study, is in line with the effects on decision dynamics of quantitative differences in numerical stimuli^22^,^46^ and learned reward differences^24^,^26^,^47^,^48^. This effect may have been exacerbated by the fact that on the first choice at a delay, novel delay information was provided for the Future option and participants may have proceeded towards the high value stimulus prior to incorporating delay information. In a further study by Calluso and colleagues^45^, initial response movements were biased towards the Future option as observed in the current study. In more detailed analyses, these researchers suggested that ‘farsighted’ participants (those who did not discount strongly) defaulted towards the larger later option, whereas more impulsive participants had less biased trajectories. It is worth noting that Calluso and colleagues presented Future options that began at 150% of the Now option and increased to 600%, so the range of amount ratios presented may have encouraged choosing later alternatives more than in the study by Dshemuchadse and colleagues. The differences across these studies highlight the complexity of intertemporal choice, which by its nature depends two different sources of information to evaluate each option^50^,^51^. Much further work is required to investigate these processes and their effects on response trajectories.

The current study employed hypothetical intertemporal decisions. Regardless of their choices, participants could not enable immediate access to or access greater amounts of the monetary incentives or vices described in the experiment. The nature of the reward (i.e. whether it is real or hypothetical) is known to impact on discounting effects^38^. While the patterns of discounting observed in the current study are, in the main, in line with effects in previous studies, no significant difference in discounting was observed across vice groups on monetary decisions, which might indicate reduced attractiveness of the hypothetical monetary rewards (reduced attractiveness of rewards may reduce discounting). In contrast, for vice decisions, participants who chose smoking as a vice engaged in significantly greater discounting than participants who chose alcohol or food. Participants who chose smoking also discounted cigarettes more strongly than money, which was not observed for the other two vice groups. So, in the current study, different effects were observed for hypothetical monetary and vice decisions.

The staircase procedure employed in the current study allowed us to investigate whether decision dynamics influenced by a specific delayed reward predicted further decisions including that delayed reward. The procedure also enabled us to estimate delay discounting effects quickly, which was important given the nature of the setting. However, one weakness of the staircase procedure is that it is possible to learn to respond strategically to “earn” greater rewards. For instance, when presented with the initial choice between 8 Now and 16 at a delay, choosing €8 Now reduced the next Now value (€4 Now), but choosing €16 at a delay increased the next Now value (€12 Now; see Fig. 1B). Consequently, choosing the delayed option is the best option if one is strategic. While it is certainly possible to respond in this way, the data suggest that such strategic performances were not common. The strategic approach remains the same regardless of delay, so the typical effects of delay on subjective value observed in the current study would have been reduced if participants employed the strategic approach. Any relationship between decision dynamics and subjective value would also likely have been reduced by strategic responding.

The current experiment was conducted in noisy conditions and under tight time constraints. Nevertheless, it was possible to identify clear relationships between decision dynamics and cognitive evaluation. We also provided real-time feedback to our participants by showing them their discounting curves immediately following participation. In our experience, taking experiments to the public is enlightening for both researchers and the public and we were able to engage the public in conversations about the importance of intertemporal choice that the public might not otherwise have thought worth their interest. The brevity of the paradigm and the environment of the Science Gallery facilitated this interaction and beneficial outcomes beyond the study.

## Method

### Setting and Recruitment

Testing took place at the Science Gallery Dublin^*TM*^, Trinity College Dublin, as part of the RISK LAB exhibition (02/05/2013 to 23/06/2013^36^). All data were collected in accordance with procedures approved by the Research Ethics Committee at the National University of Ireland Galway (http://www.nuigalway.ie/research/office_dean_research/ethics.html) and informed consent was obtained from all participants. The RISK LAB exhibition provided an interactive educational experience concerning the psychology and mathematics of everyday risks and risk-taking. It included a variety of art pieces and instructive games and, in addition, members of the visiting public were invited to take part in a range of short engaging behavioral experiments, including the current study entitled *Price your Vice*. Interested patrons were provided information on all the experiments and were asked to sign a consent form by a member of Science Gallery staff prior to participation. Patrons who registered were allocated a participation card which contained a unique ID number and asked to provide limited demographic information. It was not possible to participate in the experiments without this card. The RISK LAB experiments were presented in the centre of the gallery at a number of computer terminals arranged in a small circle with stools in front of them, much like a set of levered gambling machines (‘one-armed bandits’), which was in keeping with the gallery’s theme of risk and decision making. Patrons were continuously monitored by Science Gallery staff in case the behavioral experiments caused any offence or discomfort. No adverse reactions were reported during the two-month exhibition.

Given the optional nature of the experiment, demographic data were not obtained for all participants. Of the 625 unique participation cards registered, only 469 participants completed demographic data questionnaires. The availability of demographic data was included in all statistical models to assess whether performances of those that provided demographic data were different for those that did not. Those that completed demographic data were not different on any of the measures tested. Demographic data from the 469 recorded participants are presented in Table 7. As might be expected, the breadth of the population available was greatly increased relative to typical laboratory studies. However, it is worth noting that these participants were visitors at a science museum and therefore constitute a subset of the general adult population.

### Procedure

To begin an experiment in RISK LAB, participants placed their ID card on the game machine, which registered the ID of the user. Next, the participants pulled the side-mounted lever and, following a brief animation, they were randomly assigned to one of six behavioral experiments. Participants were allowed to complete as many experiments as they wished in any order. For the purposes of the current study, only data from a participant’s first exposure to the *Price your Vice* experiment was included. On starting the experiment (a computer program coded in Adobe Flex (R)), participants completed a short ‘warm up’ in which they used a computer mouse to click stationary squares on the computer screen and they were provided with further information specific to the experiment (i.e., the number of decisions, the purpose of the study). Following this, participants self-identified themselves as under or over 18. Those under 18 were provided with a children’s version that included computer games and social media as vices. Those that self-identified as children were excluded from the current analyses.

Participants who self-identified as over 18 were provided with a list of ‘vices’ (alcohol, cigarettes, food or “none of the above”), and asked to select the item they most craved “right now”. For participants who chose cigarettes, the vice unit was 1 cigarette, so the first decision was between 8 cigarettes Now and 16 cigarettes at a Delay. If participants chose alcohol as their vice, they were asked to make a further choice between beer, wine and cocktails to make the vice units more personalised. Those that chose beer and wine were asked to choose between bottles of their vice (e.g., 8 bottles of beer Now or 16 bottles of beer at a Delay) and those that chose cocktails chose between quantities of cocktails (e.g., 8 cocktails Now or 16 cocktails at a Delay). Participants who chose food as their vice chose chocolate or “crisps” (potato chips), and during vice decisions, made choices between quantities of bars of chocolate (e.g., 8 bars of chocolate Now or 16 bars of chocolate at a Delay) or packets of crisps (e.g., 8 packets of crisps Now or 16 packets of crisps at a Delay).

Depending on the choice of vice, participants completed one or two sets of 20 decisions. The first set of 20 decisions required the participants to choose between an amount of money available now and an amount of money at a later date. Participants who chose “none of the above” as their vice finished their participation at this point. The second set of decisions required participants to choose between an amount of their pre-selected vice available now and an amount of the vice at a later date. In the instructions, participants were made aware that they would not receive any monetary or vice-related rewards, but were requested to make each choice as if it were real. In addition, participants were requested (1), when considering the Future option, to treat it as though they were guaranteed to receive it after the specified delay (there is no risk) and (2) to consider that they would receive brand new goods at the end of the delay, not leftover goods.

Each set of 20 decisions consisted of 4 decisions at each of 5 delays (1 week, 1 month, 6 months, 1 year, 3 years) similar to Tsukayama and colleagues^35^. Delays were presented in a variety of sequences: increasing (from 1 week to 3 years), decreasing (from 3 years to 1 week) and 5 sequences from a 5 × 5 latin square, in which each delay occurs in each sequential position (1st to 5th) and exactly once before and after every other delay. Within each delay, participants began by choosing between €8 now and €16 at a delay (e.g., €8 now or €16 in a week). Following each decision, the amount of money available now adjusted in order to identify the equivalence point, the subjective value of the Future reward in terms of the amount of money now (e.g., €16 in a week might subjectively be worth close to €16 now whereas €16 in 3 years may be worth less). During each choice, the participant’s mouse cursor position was recorded at approximately 60 Hz. The x, y coordinates and timestamps were stored in a file with other decision (e.g., which stimuli were presented) and participant (e.g., ID number, vice chosen) characteristics and this file was sent to a remote computer in NUI Galway. Having completed 20 decisions assessing discounting of euro rewards, participants then completed 20 similarly designed decisions, choosing between amounts of their pre-selected vice at a variety of delays.

Visual presentation of decisions followed standard procedures in mouse-cursor tracking experiments. Screens were 23 inch widescreen monitors with a resolution of 1920 × 1080 and a refresh rate of 60Hz. As can be seen in Fig. 1, section A, two options were presented in each decision and these were located in the top left and top right of the screen. Participants clicked a “Next” button at the button of the screen to begin a decision and both options were presented simultaneously (the “Next button disappeared). Amount (e.g., €8) and delay (“Now”) information were presented simultaneously with amount above delay. Once participants clicked on one of the available options, the “Next” button at the bottom of the screen reappeared.

Following 40 decisions, participants were presented with a graph depicting their personal discounting curve for both money and their pre-selected vice. This screen provided some brief information about discounting and its relevance in understanding impulsivity, in line with the goals of the Science Gallery exhibition. Debriefing information (such as links to supportive resources) was made available participants on this screen if they chose to view it. There was no overall time limit imposed on participants but participants were prompted to complete a decision if they had not moved within 2 seconds. Science Gallery staff restarted terminals if participants left without completing participation.

### Statistical analyses

Mixed effects models were employed to assess statistical significance of effects^42^,^43^. The first set of analyses conducted for each set of decisions assessed predictors of decision dynamics. A linear model was employed to predict response time, a binomial model to predict wide or tight trajectories (based on dichotomised maximum deviation) and a poisson model to predict number of x flips. Decision trajectories with response times greater than 5 seconds or more than 10 x flips were excluded from analyses. Fixed effects in these models of indices of decision dynamics included Delay (the delay until a reward), Choose Now (whether the chosen option was the Now option or the Future option) and three control variables: Demographics, Decision Number and First Choice. Of the 625 participants who were allocated an ID card, only 429 completed the demographic questionnaires. In order to include the greatest sample size, we coded the presence or absence of demographic information to see whether those that did complete performed differently from those who provided such information. The effects of Decision Number and Delay were assessed using log transformed variables to capture the nonlinear effects of practice and delay. First Choice and Choose Now contrasts were coded as True (+0.5) and False(−0.5) to facilitate interpretation of estimates and centre these predictors to reduce collinearity. The log transformed Delay values were centred to prevent multicollinearity due to the interaction of Delay and the Choose Now variable. Collinearity estimates of the models reported were within acceptable limits. Kappa values were all below 6 and VIF (variance inflation factors) were all below 2.

In all of these models of indices of decision dynamics, participant was included as a random variable. Models of response time and x flips that included random slopes for the effect of Delay for each participant did not converge, so random slopes were also excluded from the maximum deviation model to facilitate comparisons across models. The beta values and errors in the maximum deviation model that included a random slope were very similar (within 0.01) to those reported in the text for the model without a random slope and the significant effects were the same across models.

The second set of statistical analyses assessed predictors of the equivalence point (the subjective value of the future option in terms of money now). Specifically, these models assessed whether characteristics of the motor execution of a decision as captured in decision dynamics predicted subjective values of future options and whether such contributions were additional to the prediction of subjective value by the delay until the future option. To create the outcome variable, equivalence points were converted to proportion of the subjective value of €16 Now. Fixed effects of demographics (control), the log of Delay, response time, dichotomised maximum deviation and x flips were included in the models. Separate models were employed to assess movement during choices of the Now option and choices of the Future option to simplify interpretation. If we included both Now and Future choices in the one model, the effects of response time, dichotomised maximum deviation and x flips would interact with the choice made (i.e., Now or Future), such that increases in the decision dynamics indices would predict increases in the equivalence point following a Now choice but decreases in the equivalence point following a Future choice. In addition, a majority of equivalence point values were very low (approaching €zero) or very high (approaching €16) and it was easier to assess the effects of transformations on the outcome variable when it was skewed in one direction only.

Two further analysis choices were made. In the models of equivalence points reported in the text, we employed the raw equivalence point score as the outcome variable. We also tested an arcsin transformation of the outcome variable and this provided a slightly better fit. However, it is more difficult to interpret the relationship between the beta values of the predictors and change in the outcome variable when using the arcsin transformation. The significant effects were the same for the transformed variable as those reported in the text for the untransformed variable (beta values were different because of the different outcome variable). The second analysis choice that we made was to include only a random intercept for participant and not a random slope. Similarly to the analyses of decision dynamics above, the inclusion a random slope made the model less identifiable and beta values were similar and the significant effects the same as those in the simpler model which is reported in the text.

## Additional Information

**How to cite this article**: O’Hora, D. *et al.* Decisions in Motion: Decision Dynamics during Intertemporal Choice reflect Subjective Evaluation of Delayed Rewards. *Sci. Rep.* **6**, 20740; doi: 10.1038/srep20740 (2016).

## Figures and Tables

**Table 1:** Statistical analyses of effects on decision dynamics indices during Monetary Decisions.

Predictor	Response Time (log)		Curvature [*p*(Wide)]		Vacillation (x flips)	
	*b*	*SE*	*b*	*SE*	*b*	*SE*
(Intercept)	8.1063	0.0154	−0.3897	0.0841	1.2100	0.0237
Demographics	0.0400	0.0230	0.0313	0.1061	−0.0070	0.0353
Decision No. (log)	−0.1648***	0.0048	−0.1876***	0.0313	−0.0731***	0.0077
First Choice	0.2719***	0.0082	0.4324***	0.0526	0.2318***	0.0129
Delay (log)	−0.0097	0.0021	0.0151	0.0137	−0.0049	0.0034
Choose Now	−0.0224***	0.0079	0.1800***	0.0497	0.0011	0.0128
Delay (log) by Choose Now	−0.0496***	0.0040	−0.2978***	0.0263	−0.0596***	0.0066

Demographics refers to the presence of demographic data (see Method for details) and was included as a control. Decision Number refers to numerical position of the decision within the set of monetary decisions (the minimum was 1 and maximum was 20) and it assessed the change in dynamics across consecutively presented decisions. First Choice refers to the first decision at a delay. Delay was included in the model as the proportion of total delay (i.e., 3 years) to capture the effect of delay on dynamics and Choose Now refers to whether the participants chose the Now option or the Future option. Decision Number and Delay were log transformed to capture the nonlinear relationships between these variables and movement (e.g., response time reduced more quickly across the first few decisions than across the last few).

Mixed effects models with Participant as random variable.

*p < 0.05, **p < 0.01, ***p < 0.001.

**Table 2:** Statistical analyses of relationship between movement indices and subjective value during monetary decisions.

Predictor	Now Choices				Future Choices			
	*b*	*SE*	*t*	*p* (*χ*^2^)	*b*	*SE*	*t*	*p* (*χ*^2^)
(Intercept)	0.2278	0.0066	34.5902	0.0000	0.7743	0.0073	105.8247	0.0000
Demographics	−0.0250	0.0131	−1.9057	0.0567	−0.0218	0.0142	−1.5348	0.1248
Delay (log)	−0.0440	0.0019	−23.7257	0.0000	−0.0294	0.0021	−13.7683	0.0000
Response Time (log)	0.0218	0.0038	5.7398	0.0000	−0.0013	0.0045	−0.2947	0.7682
Wide Trajectory	0.0233	0.0073	3.1743	0.0015	−0.0207	0.0091	−2.2795	0.0226
X Flips	−0.0066	0.0036	−1.8322	0.0669	−0.0043	0.0044	−0.9896	0.3224

To create the outcome variable, equivalence points were converted to proportion of the subjective value of €16 Now. Linear mixed models were employed with participant as random variable. Delay and Response Time were log transformed. In addition, to facilitate model fit, z scores of log Response Time and X Flips were used.

**Table 3:** Correlations between impulsivity *k* and all three movement indices during monetary decisions.

Delay	Choice	n	Percent	Response Time (log)		Curvature (Max. Deviation)		Vacillation (x flips)	
				*r*	*p*	*r*	*p*	*r*	*p*
1 week	Now	135	25.6	0.3095	0.0057	0.1660	0.6709	0.0584	1.0000
1 week	Future	393	74.4	0.2311	0.0001	0.1852	0.0051	0.1402	0.0914
1 month	Now	237	44.6	0.1838	0.0815	−0.1266	0.6709	0.0844	1.0000
1 month	Future	294	55.4	0.0303	1.0000	0.1437	0.2186	0.1801	0.0366
6 months	Now	322	64	−0.1876	0.0143	−0.2858	0.0000	−0.0959	0.7728
6 months	Future	181	36	0.0124	1.0000	0.3082	0.0006	0.0898	1.0000
1 year	Now	427	76.5	−0.2547	0.0000	−0.2488	0.0000	−0.1152	0.2587
1 year	Future	131	23.5	0.0202	1.0000	0.3074	0.0074	0.2020	0.2897
3 years	Now	488	88.1	−0.2020	0.0002	−0.2298	0.0000	−0.0812	0.7307
3 years	Future	66	11.9	−0.2276	0.7271	0.0567	1.0000	0.0021	1.0000
Mean	Now	547		−0.0881	0.0788	−0.2158	0.0000	−0.0297	0.4884
Mean	Future	467		0.1134	0.0428	0.1821	0.0004	0.1640	0.0015

The first column indicates the delay presented in the Future option within a choice (e.g., “1 month” refers to choices between €8 now and €16 in 1 month). Probability values were adjusted using a Holm-Bonferoni correction^55^.

**Table 4:** Statistical analyses of effects on decision dynamics measures during Vice Decisions.

Predictor	Response Time (log)		Curvature [*p*(Wide)]		Vacillation (x flips)	
	*b*	*SE*	*b*	*SE*	*b*	*SE*
(Intercept)	7.8931	0.0191	−0.6972	0.0880	1.1425	0.0259
Demographics	0.0079	0.0311	0.7951	0.0814	0.0389	0.0396
Decision No. (log)	−0.1471***	0.0053	−0.0608	0.0324	−0.0775***	0.0081
First Choice	0.2521***	0.0089	0.5555***	0.0539	0.2454***	0.0135
Delay (log)	−0.0047	0.0023	0.0252	0.0140	0.0047	0.0036
Choose Now	0.0105	0.0087	0.3268***	0.0512	0.0384***	0.0135
Delay (log) by Choose Now	−0.0445***	0.0043	−0.2637***	0.0269	−0.0596***	0.0069

Details of predictors are presented in Table 1.

Mixed effects models with Participant as random variable.

*p < 0.05, **p < 0.01, ***p < 0.001.

**Table 5:** Statistical analyses of relationship between movement indices during vice decisions and Subjective Value.

Predictor	Now Choices				Future Choices			
	*b*	*SE*	*t*	*p* (*χ*^2^)	*b*	*SE*	*t*	*p* (*χ*^2^)
(Intercept)	0.2297	0.0073	31.2922	0.0000	0.7691	0.0076	101.0416	0.0000
Demographics	−0.0249	0.0146	−1.6997	0.0892	−0.0246	0.0146	−1.6858	0.0918
Delay (log)	−0.0363	0.0021	−17.6049	0.0000	−0.0284	0.0020	−13.9261	0.0000
Response Time (log)	0.0172	0.0043	3.9770	0.0001	−0.0227	0.0040	−5.7388	0.0000
Wide Trajectory	0.0192	0.0080	2.4119	0.0159	−0.0208	0.0083	−2.5172	0.0118
X Flips	0.0079	0.0041	1.9134	0.0557	0.0047	0.0038	1.2309	0.2184

Details of predictors are presented in Table 2.

**Table 6:** Correlations between impulsivity *k* and all three movement indices during vice decisions.

Delay	Choice	n	Percent	Response Time (log)		Curvature (Max. Deviation)		Vacillation (x flips)	
				*r*	*p*	*r*	*p*	*r*	*p*
1 week	Now	115	23.1	−0.0888	1.0000	−0.1490	1.0000	−0.0657	1.0000
1 week	Future	383	76.9	0.1934	0.0031	0.2027	0.0016	0.1314	0.1708
1 month	Now	199	40.4	−0.0479	1.0000	−0.0924	1.0000	0.0608	1.0000
1 month	Future	293	59.6	0.2965	0.0000	0.2502	0.0004	0.1269	0.4183
6 months	Now	263	55.0	−0.2983	0.0000	−0.2095	0.0120	−0.2224	0.0058
6 months	Future	215	45.0	0.1408	0.4691	0.1728	0.1784	0.0449	1.0000
1 year	Now	345	65.5	−0.3017	0.0000	−0.2049	0.0029	−0.1624	0.0446
1 year	Future	182	34.5	0.1161	1.0000	0.1584	0.4252	0.0369	1.0000
3 years	Now	409	78.2	−0.3798	0.0000	−0.2452	0.0000	−0.1213	0.2120
3 years	Future	114	21.8	0.1824	0.5727	0.3284	0.0072	0.1616	0.8584
Mean	Now	463		−0.3568	0.0000	−0.2834	0.0000	−0.1345	0.0037
Mean	Future	442		0.2569	0.0000	0.2359	0.0000	0.1529	0.0025

The first column indicates the delay presented in the Future option within a choice (e.g., “1 month” refers to choices between 8 units of a vice now and 16 units of a vice in 1 month). Probability values were adjusted using a Holm-Bonferroni correction.

**Table 7:** Demographic data for 469 participants in the current study.

Age	Nationality	L1 English	Education	Gender
Min. : 15.00	Irish : 307	Yes: 389	Graduate Degree (Masters/PhD): 103	Female: 209
1st Qu.: 23.00	American: 31	No : 73	Undergraduate (Honours degree): 158	Male : 259
Median : 27.00	British : 20		Undergraduate (other): 110	
Mean : 31.31	French : 17		Post-Primary : 85	
3rd Qu.: 34.00	German : 13			
Max. : 80.00	English : 12			
	(Other) : 69	No response : 7	(Other): 13	No Response: 1
